# Health‐Related Quality of Life and Its Determinants Among Hypertensive Patients in Southwest Ethiopia: A Cross‐Sectional Study

**DOI:** 10.1002/puh2.70233

**Published:** 2026-04-13

**Authors:** Tewodros Yosef, Muluken Haile, Nigusie Shifera, Tadesse Nigussie, Binyam Girma Sisay, Molla Asnake Kebede, Hailemelekot Bekele Kebede, Alemnew Destaw, Andualem Henok

**Affiliations:** ^1^ School of Public Health College of Medicine and Health Sciences Mizan‐Tepi University Mizan Teferi Ethiopia; ^2^ School of Medicine, IMPACT The Institute for Mental and Physical Health and Clinical Translation Deakin University Geelong Victoria Australia; ^3^ Department of Public Health College of Health Sciences Salale University Fiche Ethiopia; ^4^ Department of Nutrition and Dietetics School of Public Health Addis Ababa University Addis Ababa Ethiopia; ^5^ School of Medicine College of Medicine and Health Sciences Mizan‐Tepi University Mizan Teferi Ethiopia; ^6^ School of Public Health Asrat Woldeyes Health Science Campus Debre Berhan University Debre Berhan Ethiopia

**Keywords:** health‐related quality of life, hypertension, mental component summary, physical component summary, southwest Ethiopia

## Abstract

**Background:**

Hypertension (HTN) increases mortality, healthcare costs, and disability, posing a major global public health challenge and reducing health‐related quality of life (HRQoL). Therefore, this study aimed to assess the HRQoL and its associated factors among hypertensive patients at selected hospitals in southwest Ethiopia.

**Methods:**

A cross‐sectional study conducted among 385 patients in three hospitals in southwest Ethiopia (May–July 2022) assessed HRQoL using the 36‐Item Short Form Survey (SF‐36). Physical component summary (PCS) and mental component summary (MCS) scores were calculated separately. Linear regression analysis was used to identify factors associated with the physical and mental health summaries of HRQoL, with statistical significance set at *p* < 0.05.

**Results:**

The PCS score was 70.6 ± 22.8, whereas the MCS score was 56.4 ± 12.7. Factors associated with PCS included advanced age (*β* = −0.54, *p* < 0.001), female sex (*β* = −3.69, *p* = 0.040), higher income (*β* = 0.0015, *p* = 0.001), larger family size (*β* = −2.35, *p* < 0.001), absence of comorbidity (*β* = 12.6, *p* < 0.001), Stage II HTN (*β* = −4.59, *p* = 0.047), and Stage III HTN (*β* = −15.8, *p* < 0.001). Factors associated with MCS included advanced age (*β* = −0.42, *p* < 0.001), female sex (*β* = −3.40, *p* = 0.007), higher income (*β* = 0.0001, *p* = 0.042), and Stage III HTN (*β* = −7.16, *p* = 0.023).

**Conclusion:**

Hypertensive patients had lower HRQoL across all domains. PCS and MCS scores declined with increasing age, female sex, low income, and greater HTN severity. Targeted interventions, including comorbidity management and blood pressure control, are vital to improving their quality of life.

## Graphical abstract

• Hypertensive patients had reduced quality of life, particularly in mental health.

• Older age, female sex, lower income, comorbidity, and greater hypertension severity were associated with poorer outcomes, highlighting the need for targeted interventions focusing on blood pressure control and comorbidity management.

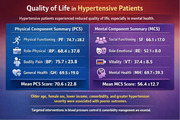

**Abbreviations**
CI: confidence interval
DBP: diastolic blood pressure
ETB: Ethiopian Birr
HRQoL: health‐related quality of life
HTN: hypertension
MCS: mental component summary
PCS: physical component summary
QoL: quality of life
SBP: systolic blood pressure
SF‐36: Short Form‐36
SD: standard deviation
WHO: World Health Organization

## 1 Introduction

A blood pressure measurement of ≥140 mm Hg for systolic blood pressure (SBP), ≥90 mm Hg for diastolic blood pressure (DBP), or the use of antihypertensive medication is defined as hypertension (HTN) [1]. HTN is one of the leading causes of mortality and morbidity worldwide. It can easily be identified by measuring blood pressure at home or in a health center and is often effectively managed with low‐cost medications [2]. HTN is well established as a major risk factor for cardiovascular disease and stroke [3]. It is more prevalent in low‐ and middle‐income countries [2]. HTN is also associated with reduced health‐related quality of life (HRQoL) [4]. HRQOL is a multidimensional concept that reflects how an individual's health status affects their overall quality of life (QoL) [5]. It is an important outcome measure when evaluating HTN treatment [6]. HRQoL is defined as an individual's or group's perceived physical and mental health (MH) over time [7].

The QoL within an individual's worldview is shaped by culture and values. It is also influenced by physical health, psychological state, level of independence, and social connections [8]. Saleem et al. [9] found that HRQoL among hypertensive patients was generally lower across all domains of the Short Form‐36 (SF‐36) compared to individuals with normal blood pressure. Low HRQoL is significantly associated with increased mortality and a greater burden on healthcare systems and society as a whole [10].

The factors affecting HRQoL among hypertensive patients are varied [6]. Sociodemographic characteristics (age, sex, marital status, education, occupation, income, and family size), clinical factors (duration of HTN as diagnosis, elevated blood pressure, number of symptoms and complications, comorbidities, number of antihypertensive drugs use and their side effects, and cost of treatment), compliance with dietary salt advice, and physical activity are predictors of HRQoL [4, 6, 9, 11, 12, 13, 14, 15, 16, 17].

Despite increased attention to HRQoL among patients with chronic conditions, health facilities remain primarily focused on treatment and are not well informed about the socioeconomic and clinical implications of these conditions [18], which represents a major barrier to improving patients’ HRQoL [19]. The identification of patient characteristics associated with HRQoL is critical for defining intervention targets that may benefit from enhanced clinical care [20].

Few studies in Ethiopia have examined HRQoL among hypertensive patients, with most focusing on Addis Ababa [21, 22, 23, 24], and only a few were conducted in the northern regions [11, 25] and the Oromia region [12]. There are no available data on HRQoL among hypertensive patients in the southwest region, particularly in the present study area. Investigating HRQoL in this area is essential, given the country's geographic diversity and variability in healthcare resources. Therefore, this study aimed to assess the HRQoL and its associated factors among hypertensive patients at selected hospitals in southwest Ethiopia.

## 2 Methods

### 2.1 Study Design, Setting, and Period

A hospital‐based cross‐sectional study was conducted at selected hospitals in southwest Ethiopia. There are three general hospitals, one teaching hospital, and 10 primary hospitals in the region. Three hospitals were randomly selected. These hospitals provide several clinical departments, including internal medicine, surgery, pediatrics, gynecology/obstetrics, dentistry, and psychiatry. The settings include a chronic follow‐up clinic that provides care for patients with diabetes, HTN, and epilepsy. The study was conducted between May 20 and July 20, 2022.

### 2.2 Populations

The source population comprised all hypertensive patients attending follow‐up clinics at the selected hospitals. The study population included those who met the inclusion criteria and voluntarily participated. The study included patients aged 18 years and older who had been receiving treatment for at least 3 months. Patients who were severely ill, unable to communicate, or unwilling to provide consent were excluded.

### 2.3 Sample Size Calculation

The sample size was determined using a single population mean formula, on the basis of HRQoL scores among hypertensive patients (64.8 ± 19 standard deviation [SD]) [11], assuming a 95% confidence level and a margin of error (precision) of 3 units:

$$n=\frac{Z^{2}\sigma^{2}}{E^{2}}=\frac{{\left(1.96\right)}^{2}{\left(19\right)}^{2}}{3^{2}}=155$$

The final sample size was 427 after accounting for a design effect of 2.5 and a 10% non‐response rate.

### 2.4 Sampling Procedures

Out of the 14 hospitals in the southwest region, three were randomly selected. The sample size for each hospital was determined by the number of patients with HTN attending follow‐up clinics. Patients with HTN attended monthly checkups unless emergencies occurred. Participants were selected using systematic random sampling. The sampling interval (*K* = *N*/*n*) was calculated, and the first participant was chosen randomly using a lottery method. Subsequently, every *K*th participant was included until the sample size was reached, with replacements made for ineligible individuals or those who declined to participate.

### 2.5 Data Collection Tools, Procedures, and Quality Control

HRQoL was assessed using the standardized SF‐36 tool [26], which has been validated for use in the local context [27, 28]. The SF‐36 is a widely recognized tool for assessing general HRQoL, covering both mental and physical health. It consists of eight subscales: physical functioning (PF), role‐physical limitations (RP), bodily pain (BP), general health (GH), vitality (VT), social functioning (SF), role‐emotional limitations (RE), and MH. These subscales are combined to generate two composite scores representing physical and MH components [26]. The physical component summary (PCS) is calculated from PF, role limitations due to physical problems, BP, and GH subscales. The mental component summary (MCS) is derived from VT, SF, role limitations due to emotional problems, and MH subscales. In addition to the SF‐36 questionnaire to measure HRQoL, the survey included questions about participants’ sociodemographic and clinical characteristics. Sociodemographic and some clinical data were collected through interviews, whereas additional clinical information was obtained from patients’ medical records. The questionnaire was originally prepared in English, translated into Amharic, and then retranslated back into English to ensure data quality. A pre‐test was conducted on 5% of patients with HTN at health facilities not included in the study. Supervisors and data collectors received 2 days of training on data collection procedures and measurement tools.

### 2.6 Study Variables and Measurements

The outcome variable was HRQoL (PCS and MCS). Sociodemographic characteristics (age, sex, marital status, religion, education, occupation, average monthly family income, and family size) and clinical characteristics (number of symptoms, time since diagnosis, current blood pressure status, and the presence of comorbidities) were the independent variables.

HTN classification was defined as follows: SBP < 120 mm Hg and DBP < 80 mm Hg were considered normal; SBP 120–129 mm Hg and DBP < 80 mm Hg were considered elevated; SBP 130–139 mm Hg or DBP 80–89 mm Hg were considered Stage I HTN and SBP ≥ 140 mm Hg or DBP ≥ 90 mm Hg were considered Stage II HTN. Stage III HTN was defined as SBP ≥ 180 mm Hg or DBP ≥ 120 mm Hg [29]. HTN symptoms were defined as patient‐reported symptoms, such as headaches, fatigue, blurred vision, frequent insomnia, dizziness, and facial flushing [30]. Comorbidity was defined as the presence of any disease other than HTN reported by the patient.

### 2.7 Statistical Analysis

Descriptive statistics were used to summarize participant characteristics and covariates associated with self‐reported HRQoL. All continuous variables were normally distributed and thus summarized using the mean and SD. Categorical variables were reported as frequencies and percentages. Potential covariates were selected on the basis of theory and prior research and then screened using bivariable analysis. Variables with *p* < 0.25 were included in the multivariable model to retain important predictors while reducing overfitting. The assumptions of linear regression were checked: linearity via scatterplots of continuous predictors versus the outcome, homoscedasticity using residuals versus fitted values plots, normality of residuals using *Q*–*Q* plots and the Shapiro–Wilk test, and multicollinearity using variance inflation factors (all <2). No significant interactions between predictors were detected, and all assumptions were adequately met. Linear regression analysis was used to identify variables associated with the physical and MH summaries of HRQoL. Adjusted *β* coefficients with their 95% confidence intervals are reported, with statistical significance set at *p* < 0.05. The data were analyzed using Stata version 18 (Stata Corp. 2023. Stata Statistical Software: Release 18. College Station, TX, USA: Stata Corp LLC).

### 2.8 Ethical Approval

Ethical approval was obtained from the Mizan‐Tepi University Research and Ethics Committee (PGC/079/2022). Privacy and confidentiality were upheld throughout the data collection, analysis, and reporting processes, with information gathered from respondents shared only with the lead investigator and data collectors. Written informed consent was obtained from each participant after a clear explanation of the study.

## 3 Results

### 3.1 Sociodemographic Characteristics

The response rate was 90.2%, with 385 out of 427 participants completing the interview. Of these, 219 (56.9%) were male, and 232 (60.2%) were married. Sixty‐six percent of the participants were urban dwellers (Table 1). The mean age was 53.3 ± 11.9, ranging from 28 to 86 years. The average family size was 3.94 ± 2.19, ranging from 1 to 15, with the majority (326, 84.7%) having a family size of fewer than five members. The average family monthly income was 4457.5 ± 2279.9 Ethiopian Birr (ETB), ranging from 1553.9 to 10,350.7 ETB.

**TABLE 1:** Sociodemographic characteristics of the participants at selected hospitals in southwest Ethiopia.

Variables	Categories	Frequency	Percent
Sex	Male	219	56.9
	Female	166	43.1
Marital status	Married	232	60.2
	Single	33	8.6
	Widowed/widower	75	19.5
	Divorced	45	11.7
Religion	Orthodox	163	42.3
	Protestant	104	27.0
	Catholic	40	10.4
	Muslim	76	19.8
	Other	2	0.5
Residence	Urban	254	66.0
	Rural	131	34.0
Occupation	Farmer	54	14.0
	Merchant	93	24.2
	Daily laborer	3	0.8
	Govt. employee	99	25.7
	Housewife	90	23.3
	Student	46	11.9
Monthly income, ETB	4457.5 ± 2279.9		

Abbreviation: ETB, Ethiopian Birr.

### 3.2 Clinical Characteristics

Among the 385 participants, 66.5% had Stage I HTN, 28.6% had Stage II, and 4.9% had Stage III HTN. Headache was the most common symptom, occurring in 42.5% of participants. Approximately 10.9% had been diagnosed with HTN for over 10 years, with an average duration of 4.7 ± 3.1 years since diagnosis. Nearly 21% had a comorbidity (Table 2).

**TABLE 2:** Clinical characteristics of the participants at selected hospitals in southwest Ethiopia.

Variables	Categories	Frequency	Percent
Current blood pressure status	Stage I	256	66.5
	Stage II	110	28.6
	Stage III	19	4.9
Duration since diagnosis for the first time (4.7 ± 3.1 years)	<5 years	195	50.6
	5–9 years	148	38.4
	≥10 years	42	10.9
Hypertension‐related symptoms	Headache	164	42.5
	Fatigue	79	20.5
	Blurring of vision	50	13.0
	Insomnia	26	6.8
	Dizziness	38	9.9
	Facial flushing	28	7.3
Comorbidity	Yes	81	21.0
	No	304	79.0

### 3.3 Domains of HRQoL

The average scores were as follows: PF (74.7 ± 28.2), RP limitation (68.4 ± 37.8), RE limitation (52.1 ± 8.0), VT (37.4 ± 8.5), MH (69.7 ± 39.3), SF (66.1 ± 17.0), BP (75.7 ± 23.8), and GH (69.5 ± 19.0). The mean PCS score was 70.6 ± 22.8, whereas the mean MCS score was 56.4 ± 12.7 (Table 3).

**TABLE 3:** The mean and standard deviation of the health‐related quality of life (HRQoL) domain scores among participants at selected hospitals in southwest Ethiopia.

Domains	Composite questions on SF‐36	Mean	SD
Physical functioning (PF)	3, 4, 5, 6, 7, 8, 9, 10, 11, 12	74.7	28.2
Physical role limitations (RP)	13, 14, 15, 16	68.4	37.8
Emotional role limitations (RE)	17, 18, 19	52.1	8.0
Vitality (VT)	23, 27, 29, 31	37.4	8.5
Mental health (MH)	24, 25, 26, 28, 30	69.7	39.3
Social functioning (SF)	20, 32	66.1	17.0
Bodily pain (BP)	21, 22	75.7	23.8
General health (GH)	1, 2, 33, 34, 35, 36	69.5	19.0
PCS	Mean (PF, RP, BP, and GH)	70.6	22.8
MCS	Mean (SF, RE, VT, and MH)	56.4	12.7

Abbreviations: MCS, mental component summary; PCS, physical component summary; SD, standard deviation; SF‐36, Short Form‐36.

### 3.4 Factors Associated With the PCS and MCS Scores

After conducting a multiple linear regression analysis, the PCS was found to decline by 0.54 for every 1‐year increase in age (*β* = −0.54, 95% CI: −0.72, −0.37, *p* < 0.001). Being female was associated with a 3.69‐U decline in the PCS (*β* = −3.69, 95% CI: −7.73, −0.19, *p* = 0.040). For every 1000‐ETB increase in average income, the PCS increased by 1.5 U (*β* = 1.50, 95% CI: 0.60, 2.00, *p* = 0.001). The PCS was estimated to decrease by 2.35 U for every additional family member (*β* = −2.35, 95% CI: −3.24, −1.46, *p* < 0.001). Participants with no comorbidities had a PCS that was 12.6 U higher (*β* = 12.6, 95% CI: 7.67, 17.5, *p* < 0.001). Compared to Stage I HTN, patients with Stage II HTN had a 4.59‐U lower PCS (*β* = −4.59, 95% CI: −9.10, −0.07, *p* = 0.047), whereas patients with Stage III HTN had a 15.8‐U lower PCS (*β* = −15.8, 95% CI: −23.3, −8.32, *p* < 0.001) (Table 4).

**TABLE 4:** Factors associated with physical component summary (PCS) among participants at selected hospitals in southwest Ethiopia.

Coefficients									
Model		Unstandardized coefficients		Standardized coefficients		Sig.	95% confidence interval for *β*		Collinearity statistics
		*β*	Std. error	*β*	Robust std. error		Lower bound	Upper bound	VIF
Age^**^		−1.02	0.08	−0.54	0.089	<0.001	−0.72	−0.37	1.52
Sex (*R* ^#^ = Male)^*^		−4.66	2.34	−3.69	1.92	0.040	−7.73	−0.19	1.14
Income^**^		0.002	0.0005	0.0015	0.0004	0.001	0.0006	0.002	1.13
Residence (*R* ^#^ = Urban)		1.00	2.46	1.93	1.81	0.287	−1.63	5.50	1.29
Family size^**^		−4.88	0.47	−2.35	0.45	<0.001	−3.24	−1.46	1.37
Comorbidity (*R* ^#^ = Yes)^**^		23.6	2.59	12.6	2.51	<0.001	7.67	17.5	1.17
Current BP status (*R* ^#^ = Stage I)	Stage II^**^	−13.2	2.29	−4.59	2.30	0.047	−9.10	−0.07	1.16
	Stage III^**^	−46.0	4.78	−15.8	3.81	<0.001	−23.3	−8.32	1.46
Constant		70.6	1.16	95.6	6.05	<0.001	83.7	107.5	1.28

*Note:* Dependent variable: computed PCS = mean (PF, RP, BP, and GH), *R* ^#^ = reference value.

Abbreviation: BP, bodily pain; Std., standard.

* *p* < 0.05.

** *p* < 0.01.

After running a multiple linear regression analysis, the MCS was shown to decline by 0.42 for every 1‐year increase in age (*β* = −0.42, 95% CI: −0.54, −0.31, *p* < 0.001). For every 1000‐ETB increase in average income, the MCS increased by 0.10 U (*β* = 0.10, 95% CI: 0.07, 0.40, *p* = 0.042). Being female was associated with a 3.40‐U decline in the MCS (*β* = −3.40, 95% CI: −5.58, −0.92, *p* = 0.007). Compared to Stage I HTN, patients with Stage III HTN had a 7.16‐U reduction in the MCS (*β* = −7.16, 95% CI: −13.3, −1.01, *p* = 0.023) (Table 5).

**TABLE 5:** Factors associated with mental component summary (MCS) among participants at selected hospitals in southwest Ethiopia.

Coefficients									
Model		Unstandardized coefficients		Standardized coefficients		Sig.	95% confidence interval for *β*		Collinearity statistics
		*β*	Std. error	*β*	Robust std. error		Lower bound	Upper bound	VIF
Age^**^		−0.52	0.05	−0.42	0.06	<0.001	−0.54	−0.31	1.52
Sex (*R* ^#^ = Male)		−2.16	1.30	−3.40	1.26	0.007	−5.88	−0.92	1.14
Income		0.0003	0.0003	0.0001	0.0003	0.042	0.00007	0.0004	1.13
Residence (*R* ^#^ = Urban)		0.05	1.37	0.74	1.22	0.545	−1.66	3.13	1.29
Family size^*^		−1.91	0.28	−0.60	0.33	0.069	−1.25	0.05	1.37
Comorbidity (*R* ^#^ = Yes)		6.73	1.55	1.08	1.79	0.546	−2.44	4.61	1.17
Current BP status (*R* ^#^ = Stage I)	Stage II^*^	−4.21	1.35	−0.47	1.53	0.758	−3.49	2.54	1.16
	Stage III^**^	−20.7	2.82	−7.16	3.13	0.023	−13.3	−1.01	1.46
Constant		56.4	0.65	82.6	4.10	<0.001	74.5	90.6	1.28

*Note:* Dependent variable: computed MCS = mean (SF, RE, VT, and MH), *R* ^#^ = reference value.

Abbreviation: BP, bodily pain; Std., standard.

* *p* < 0.05.

** *p* < 0.01.

## 4 Discussion

This study assessed HRQoL and its associated factors among hypertensive patients in selected hospitals in southwest Ethiopia. Overall, domain scores varied, with relatively higher scores observed for PF and BP, and lower scores for VT and role emotional domains. Compared with Adamu et al. [25], HRQoL scores in this study were generally higher; however, this contrasts with Jufar et al. [11], who reported comparable physical and mental summary scores and lower values in PF and GH. Consistent with studies in Ethiopia [12], VT was the lowest scoring domain within the MH component, suggesting that reduced energy and fatigue may be a common concern among hypertensive patients. Similarly, Muyisa et al. [31] found that hypertensive patients generally had low QoL, with over half demonstrating poor medication adherence, whereas higher adherence was associated with improved physical, social, and MH, highlighting the critical role of adherence and effective HTN management.

The mean PCS score was higher than the MCS score, indicating relatively better perceived physical health. However, this finding differs from some Ethiopian studies [11, 12], where MH scores were similar or higher. This discrepancy may reflect differences in study populations, including variation in disease severity, healthcare access, and comorbidity burden. Cultural perceptions of health may also influence how individuals report physical versus mental well‐being. Physical symptoms are often more readily recognized and reported, whereas MH concerns may be underreported due to stigma or limited awareness. Additionally, differences in the measurement context or the administration of the SF‐36 tool may contribute to these inconsistencies, underscoring the importance of considering contextual and methodological factors when interpreting HRQoL outcomes in hypertensive populations.

### 4.1 Physical Component Summary

Age was inversely associated with PCS score, consistent with findings from Ghana [6], Pakistan [9], Nepal [13], Vietnam [15], and China [4]. This relationship likely reflects age‐related functional decline and the cumulative burden of multimorbidity [32], both of which disproportionately affect the physical dimension of HRQoL. Female participants also had lower PCS scores compared to males, in agreement with previous studies [12, 33]. This difference may be partially explained by a higher burden of chronic conditions, pain, and functional limitations among women, as well as socioeconomic disadvantages that can affect access to healthcare and health resources [34]. However, as these factors were not directly measured in this study, this interpretation should be considered with caution.

Income demonstrated a positive association with PCS, supporting findings from Ethiopia [12], Pakistan [9], Nepal [13], and China [4, 17]. Greater financial resources may facilitate access to healthcare, adequate nutrition, and healthier living conditions, thereby improving physical health outcomes [17, 35, 36]. In contrast, larger family size was significantly associated with lower PCS, consistent with Ghimire et al. [13]. This relationship likely reflects the distribution of limited household resources across more individuals, which may reduce access to healthcare and constrain health‐promoting behaviors. Increased caregiving and financial demands within larger households may further limit attention to individual health needs, contributing to poorer physical outcomes [13].

The absence of comorbidities was strongly associated with higher PCS scores, in line with studies from Ethiopia [12], Vietnam [15], and China [17]. Comorbid conditions increase overall disease burden and functional limitation, thereby reducing physical health. Similarly, advanced stages of HTN were associated with progressively lower PCS scores, consistent with findings from Ethiopia [11], China [17], and India [14]. This is likely due to the increased risk of complications, such as cardiovascular events, which negatively affect PF and overall health status [16].

### 4.2 Mental Component Summary

Age was negatively associated with MCS, consistent with studies conducted in Pakistan [9], Nepal [13], and Chana [4]. This may reflect increased vulnerability to psychological conditions, including depression and anxiety, as well as cognitive decline with advancing age [32, 37]. Female participants also had lower MCS scores than males, in agreement with findings from Ethiopia [12] and India [14]. Although previous literature suggests that women may experience higher levels of psychological distress [38, 39], this study did not directly assess these mechanisms, and such interpretations should be made with caution.

Income was positively associated with MCS, consistent with prior studies conducted in Ethiopia [12], Pakistan [9], Nepal [13], and China [4, 17]. Lower income is associated with lower MCS scores [35] due to increased stress and anxiety, limited access to healthcare [17], lower QoL, higher health risks, and fewer social supports. Financial constraints can exacerbate MH issues [36], leading to reduced MCS scores. In contrast, advanced HTN (Stage III) was associated with lower MCS scores, as reported in Ethiopia [11], India [14], and China [17]. The psychological burden of living with severe disease, including concerns about complications and treatment demands, may contribute to reduce MH [11, 14].

In summary, both physical and mental components of HRQoL were influenced by demographic, socioeconomic, and clinical factors, although the nature of these associations differed. The observed inconsistencies with previous Ethiopian studies underscore the need to consider contextual, cultural, and methodological factors when interpreting HRQoL findings. Although plausible explanations are provided, particularly for sex differences and MH outcomes, these should be viewed as tentative, as the underlying mechanisms were not directly measured. Future research incorporating longitudinal designs and psychosocial variables is recommended to better elucidate these relationships.

## 5 Strength and Limitations

This study has several strengths, including the use of systematic random sampling across multiple hospitals, which enhances representativeness. It applied the validated SF‐36 tool to ensure reliable and comparable HRQoL measurement. Data were collected through interviews and medical record reviews to improve accuracy, and quality control measures, such as questionnaire pre‐testing, were implemented.

Despite its strengths, this study has some limitations. The cross‐sectional design limits causal inference, and the hospital‐based setting restricts generalizability to community populations, particularly those not attending follow‐up clinics. Some variables, including symptoms and comorbidities, were self‐reported and may be subject to recall and social desirability bias. The analysis may also be affected by residual confounding due to unmeasured factors, such as lifestyle factors (e.g., diet, physical activity, smoking, alcohol use, and medication adherence). In addition, detailed information on comorbidity types was not collected, and the length of the SF‐36 questionnaire may have contributed to respondent fatigue.

## 6 Conclusion

Hypertensive individuals in this study had lower HRQoL scores across all domains. PCS scores were lower among those of older age, female sex, lower income, larger family size, the presence of comorbidities, and those with Stage II and III HTN. Similarly, MCS scores were lower among individuals of older age, female sex, lower income, and those with Stage III HTN. These findings highlight the need for targeted interventions focusing on modifiable factors, particularly effective comorbidity management and optimal HTN control, to improve the HRQoL of hypertensive individuals.

## Author Contributions

**Tewodros Yosef**: conceptualization, methodology, software, validation, formal analysis, investigation, data curation, writing – original draft, writing – review and editing. **Muluken Haile**: conceptualization, methodology, software, validation, formal analysis, investigation, data curation, writing – original draft, writing – review and editing. **Nigusie Shifera**: visualization, formal analysis, project administration, supervision, resources, funding acquisition, writing – review and editing. **Binyam Girma Sisay**: visualization, formal analysis, project administration, supervision, resources, funding acquisition, writing – review and editing. **Molla Asnake Kebede**: visualization, formal analysis, project administration, supervision, resources, funding acquisition, writing – review and editing. **Hailemelekot Bekele Kebede**: visualization, formal analysis, project administration, supervision, resources, funding acquisition, writing – review and editing. **Alemnew Destaw**: visualization, formal analysis, project administration, supervision, resources, funding acquisition, writing – review and editing. **Andualem Henok**: visualization, formal analysis, project administration, supervision, resources, funding acquisition, writing – review and editing. All authors read and approved of the final manuscript.

## Funding

The authors have nothing to report.

## Consent

The authors have nothing to report.

## Conflicts of Interest

The authors declare no conflicts of interest.

## Data Availability

The dataset used in this study is available from the corresponding author upon reasonable request.
